# Case Report: Two Families With *HPDL* Related Neurodegeneration

**DOI:** 10.3389/fgene.2022.780764

**Published:** 2022-02-09

**Authors:** Ieva Micule, Baiba Lace, Nathan T. Wright, Nicolas Chrestian, Jurgis Strautmanis, Mikus Diriks, Janis Stavusis, Dita Kidere, Elfa Kleina, Anna Zdanovica, Nataly Laflamme, Nadie Rioux, Samarth Thonta Setty, Sander Pajusalu, Arnaud Droit, Monkol Lek, Serge Rivest, Inna Inashkina

**Affiliations:** ^1^ Latvian Biomedical Research and Study Centre, Riga, Latvia; ^2^ Children’s Clinical University Hospital, Riga, Latvia; ^3^ Centre de recherche CHU de Québec, Laval University, Québec, QC, Canada; ^4^ Department of Chemistry and Biochemistry, James Madison University, Harrisonburg, VA, United States; ^5^ Department of Pediatric Neurology, Pediatric Neuromuscular Disorders, Centre Mère Enfant Soleil, Laval University, Québec, QC, Canada; ^6^ Department of Clinical Genetics, United Laboratories, Tartu University Hospital, Tartu, Estonia; ^7^ Department of Clinical Genetics, Institute of Clinical Medicine, Faculty of Medicine, University of Tartu, Tartu, Estonia; ^8^ Department of Genetics, Yale University School of Medicine, New Haven, CT, United States

**Keywords:** spastic paraplegia, ataxia, citrate-synthase, mitochondrial diseases, brain diseases

## Abstract

There are recent reports of associations of variants in the *HPDL* gene with a hereditary neurological disease that presents with a wide spectrum of clinical severity, ranging from severe neonatal encephalopathy with no psychomotor development to adolescent-onset uncomplicated spastic paraplegia. Here, we report two probands from unrelated families presenting with severe and intermediate variations of the clinical course. A homozygous variant in the *HPDL* gene was detected in each proband; however, there was no known parental consanguinity. We also highlight reductions in citrate synthase and mitochondrial complex I activity detected in both probands in different tissues, reflecting the previously proposed mitochondrial nature of disease pathogenesis associated with *HPDL* mutations. Further, we speculate on the functional consequences of the detected variants, although the function and substrate of the HPDL enzyme are currently unknown.

## Introduction

Recently, several reports have linked the *HPDL* gene to a wide clinical spectrum of neurodegenerative phenotypes. The reported probands presented with symptoms ranging from neonatal encephalopathy to adolescent-onset uncomplicated spastic paraplegia (Ghosh et al., 2020; Husain et al., 2020; Wiessner et al., 2021). Additionally, mutations of *HPDL* were demonstrated to cause a cerebral palsy phenotype in a kindred with several affected members that had previously been attributed to variants in *GAD1* (Morgan et al., 2021).

The function of the HPDL protein is not yet known. The *HPDL* gene is paralogous to *HPPD* (which encodes 4-hydroxyphenylpyruvate dioxygenase); however, no functional association with this enzyme has been detected. The two proteins have different tissue expression patterns and localize to different subcellular compartments. *HPDL* has a wide tissue expression, with the highest levels in brain, and localizes within mitochondria (Ghosh et al., 2020; Husain et al., 2020). Studies of oxidative phosphorylation (OXPHOS) complexes from several probands with *HPDL* variants have generated inconsistent results, with decreased activity in two of five specimens from skeletal muscle, but normal activity in four proband fibroblast specimens (Husain et al., 2020).

Here, we further delineate the variable clinical course related to dysfunction of *HPDL* by characterizing two new probands, and speculate on the functional consequences of the detected variants.

## Methods

### Recruitment of Families and Ethics Statement

Persons affected with rare unidentified inherited disease have been recruited for inclusion in the Genome Database of Latvian Population (Riga, Latvia) under the framework of the Latvian Research Council project No: lzp-2018/1-0180 “The characterization and analysis of mitochondrial DNA mutations and variants of unknown significance using transmitochondrial cytoplasmic hybrid cell models”. Central Committee of Medical Ethics of Latvia approval (protocol No. 2019-3, chapter 7, from 30.05.2019.) covers all consent and data handling related issues for genetic research into the probands involved. Persons affected with rare neuromuscular, neurodegenerative, metabolic, or poly-malformation syndromes have been recruited in an interdisciplinary research program designated “Programme de Recherche et Innovation Sur les Maladies rarES” (PRISMES) at the CHU de Québec—Université Laval (CHUQC-UL) Research Centre. Research ethics board approval of the study design was obtained from the Comité d’éthique à la recherché (CER) du CHUQC-UL.

All participants and/or their legal guardians provided written informed consent prior to enrolment. The parents of the proband 1 have specifically consented to the use of video files for publication.

Complete description of methods used in DNA analysis and molecular dynamic simulations is included as Supplementary Material.

## Case Descriptions

### Proband 1

Proband 1 is a boy of 11 years old who was born to healthy unrelated parents of Latvian ethnicity. Labor was induced after 42 gestational weeks, as it did not start spontaneously. The boy first presented at 6 weeks of age, due to hypertonia, as well as partial motor and vegetative seizures, which were controlled by valproic acid after 8 days. Head magnetic resonance imaging (MRI) was conducted on brain, which revealed focal areas of delayed myelination and subcortical T1 signal changes in temporal and frontal lobes that were attributed to perinatal hypoxia. Electroencephalography (EEG) showed mild focal epileptiform activity and he was treated with valproic acid for 1 year. He has not had seizures since and does not use any antiepileptic medication. At birth, he also had unilateral cryptorchidism, which was corrected at 13 months.

His further motor and mental development were mildly delayed; he started walking at 18 months, and he spoke about 10 words at the age of 2. At 4 years, increased clumsiness was noted. At 6 years, his gait progressively deteriorated, his parents noted some cognitive decline, and he was referred for further investigations. IQ test result was 70. He had a sloping forehead and his head circumference was 49.5 cm (–1.8 SD). Neurological examination revealed spastic gait, hyperreflexia, intention tremor, dysarthria, and intermittent enuresis, and he was diagnosed with hereditary spastic paraplegia. During the next year, he developed dysphonia, dysmetria, and gait ataxia. He lost ambulation at the age of 7.5 years.

Currently, he demonstrates horizontal gaze paresis and saccadic dysmetria. Speech is characterized by dysphonia and mild scanning. He has hypomimic facial features and a slight pronator drift right > left. Muscle strength in his arms is decreased (grade 4) in the triceps and palmar interossei. Deep paresis of the legs is present; muscles are markedly hypotrophic, with grade 2 strength in almost all muscle groups. Tendon reflexes are high, with increased reflexogenic zones and positive pathological reflexes. He has bilateral ankle clonus of >10 beats and a spontaneous Babinski sign. Hand coordination testing revealed intention tremor and dysmetria. He also has truncal ataxia and can only stand and sit with support, despite retained axial muscles, with a feeling of instability in the supine position. Videos of neurological examination are included as Supplementary Material.

**FIGURE 1:**
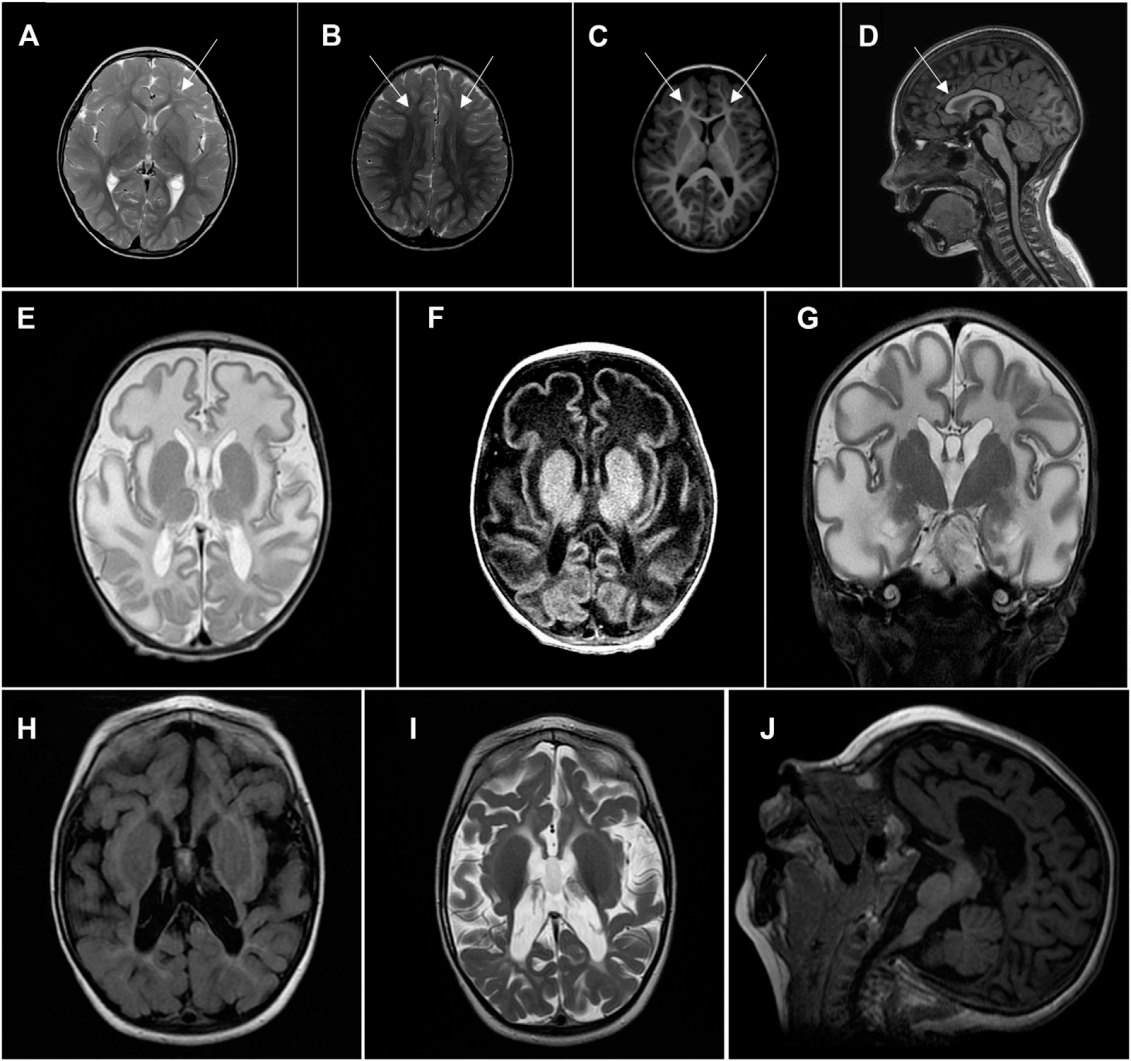
Head MRI.**(A–D)** Proband 1, 6 years of age. **(A)**, **(B)**—axial T2, frontal subcortical hyperintensity. **(C)**—axial T1, white matter reduction in frontal lobes. **(D)**—sagittal T1, smaller anterior parts of corpus callosum. **(E–G)** Proband 2, 5 weeks of age, showing significant diffuse white matter abnormalities sparing basal ganglia. **(E)**—axial T2, **(F)**—axial T1, **(G)**—coronal T2. **(H–J)** Proband 2, 2 years of age, showing a diffuse severe cotico-subcortical atrophy with white matter abnormalities but with progression of myelination. **(H)**—axial T2 flair, **(I)**—axial T2, **(J)**—sagittal T1.

Tests of biochemical indicators of mitochondrial dysfunction during different investigation periods were not suggestive of mitochondrial disease: he had normal blood lactate and alanine levels, and an unremarkable urinary organic acid spectrum. On MRI spectroscopy, lactate peak was not increased. After detection of *HPDL* variants, his brain MRI data were reevaluated, considering the MRI findings from previous reports of probands with *HPDL* variants. They showed reduction of frontal white matter, T2 frontal subcortical hyperintensities, and smaller anterior parts of the corpus callosum (Figures 1A–D). Analysis of mitochondrial respiratory chain complexes in peripheral blood leukocytes showed pronounced reduction of citrate synthase and low complex I and complex IV enzyme activities (Supplementary Figure S1). No deficiency, but rather upregulation, of complexes II and III was seen after correction for citrate synthase activity.

### Proband 2

Proband 2 is a 4-year-old boy of French Canadian ethnicity, born after a pregnancy complicated by abuse of drugs. The labor was prolonged and concluded by Cesarean section, but Apgar scores were 9-9. Disease onset was on the second day of life, with infantile spasms, which later changed to absences and tonic seizures. The seizures were refractory to treatment; currently, the proband receives three anticonvulsive medications and a moderate dose of baclofen.

Sleep apnea, apparently of central origin, was registered at 2 months of age, accompanied by sinus bradycardia. Disease progression led to the development of microcephaly at 6 months of age, his height followed the 0.1 percentile, and a G-tube was installed, due to feeding difficulties. He has severe global developmental delay, with spastic quadriplegia and dystonia. On examination, he shows normal eye movements during oculocephalic reflexes, but has no eye contact. He demonstrates sustained and spontaneous clonus, which is more pronounced on the right side. A dystonic component was observed, with increased stiffness on stimulation, axial more than peripheral. He reacts to sound, but has never attained any words, and has no purposeful movements. There have been recurrent bouts of anemia secondary to malnutrition. Scoliosis and hip luxation developed during the second year, and he also has mild elbow and knee contractures.

His blood lactic acid levels have been repeatedly elevated (2.6–4.4 mmol/L). Brain MRI was conducted twice: at 5 weeks of age, it showed significant diffuse white matter abnormalities, sparing basal ganglia, and elevated lactate peak on MR spectroscopy, supporting mitochondrial disease as a cause of his symptoms; and at 2 years of age, there was severe cortico-subcortical atrophy, with white matter abnormalities, but progression of myelination (Figures 1E–J).

Muscle biopsy showed myopathic changes, with predominant atrophy of oxidative fibers, accumulation of free glycogen, and capillary endothelium edema. Mitochondrial respiratory chain enzyme analysis in skin fibroblasts showed only reduced citrate synthase activity, with no deficiency of OXPHOS complexes detected before or after correction for citrate synthase activity. Mitochondrial respiratory chain enzyme analysis in a muscle biopsy showed complex I reduction to 43% of mean, which was not diagnostic for deficiency. Citrate synthase activity was mildly reduced in muscle biopsy cells.

## *HPDL* Variants

Whole genome sequencing of proband 1 revealed a homozygous *HPDL* (NM_032756.2) variant, c.599del (p. Gly200Alafs*4). The variant was classified as pathogenic according to ACMG interpretation guidelines (Richards et al., 2015) based on the nature of the variant—frame-shift variant in a gene for which loss-of-function variants are known mechanism of disease, the rarity of variant in population databases—6 heterozygous alleles out of 208,298 individuals and no homozygous individuals in GnomAD (Karczewski et al., 2020), and based on pathogenic prediction score of *in-silico* algorithm Cadd (Kircher et al., 2014). Both parents were confirmed to be heterozygous carriers of the variant. There was no known parental consanguinity and estimation of the inbreeding coefficient by VCFtools (Danecek et al., 2011) from next generation sequencing (NGS) data did not reveal any significant deviation, inbreeding coefficient F = 0.01. The variant transcript is not expected to be subject to nonsense mediated decay (NMD), as *HPDL* is a single-exon gene, which is invisible to the exon junction complex-dependent NMD pathway; consequently, it is likely to be translated into a truncated protein species with impaired function, due to either loss of active sites or decreased protein stability (Ghosh et al., 2020). Truncation of *HPDL* at residue 200 will lead to a loss of two of three iron binding sites, and most of the second vicinal oxygen chelate domain.

Whole exome sequencing of proband 2 identified a homozygous variant, c.1013T > C p. (Leu338Pro), in the *HPDL* gene, which was inherited from the heterozygous parents. The variant was classified as likely pathogenic according to ACMG interpretation guidelines based on the rarity of variant in population databases—7 heterozygous alleles out of 282,782 individuals and no homozygous individuals in GnomAD, the fact that the variant has been detected before in a proband with a *HPDL*-related disease (Wiessner et al., 2021), and its localization just before the iron binding site, Glu339, closest to the C-terminus of the protein (The UniProt Consortium, 2021). As well as 10 pathogenic predictions from different *in silico* algorithms BayesDel, addAF, DANN, DEOGEN2, EIGEN, FATHMM-MKL, M-CAP, MVP, MutationAssessor, MutationTaster and SIFT versus 2 benign predictions from LIST-S2 and PrimateAI (Kopanos et al., 2019).

**FIGURE 2:**
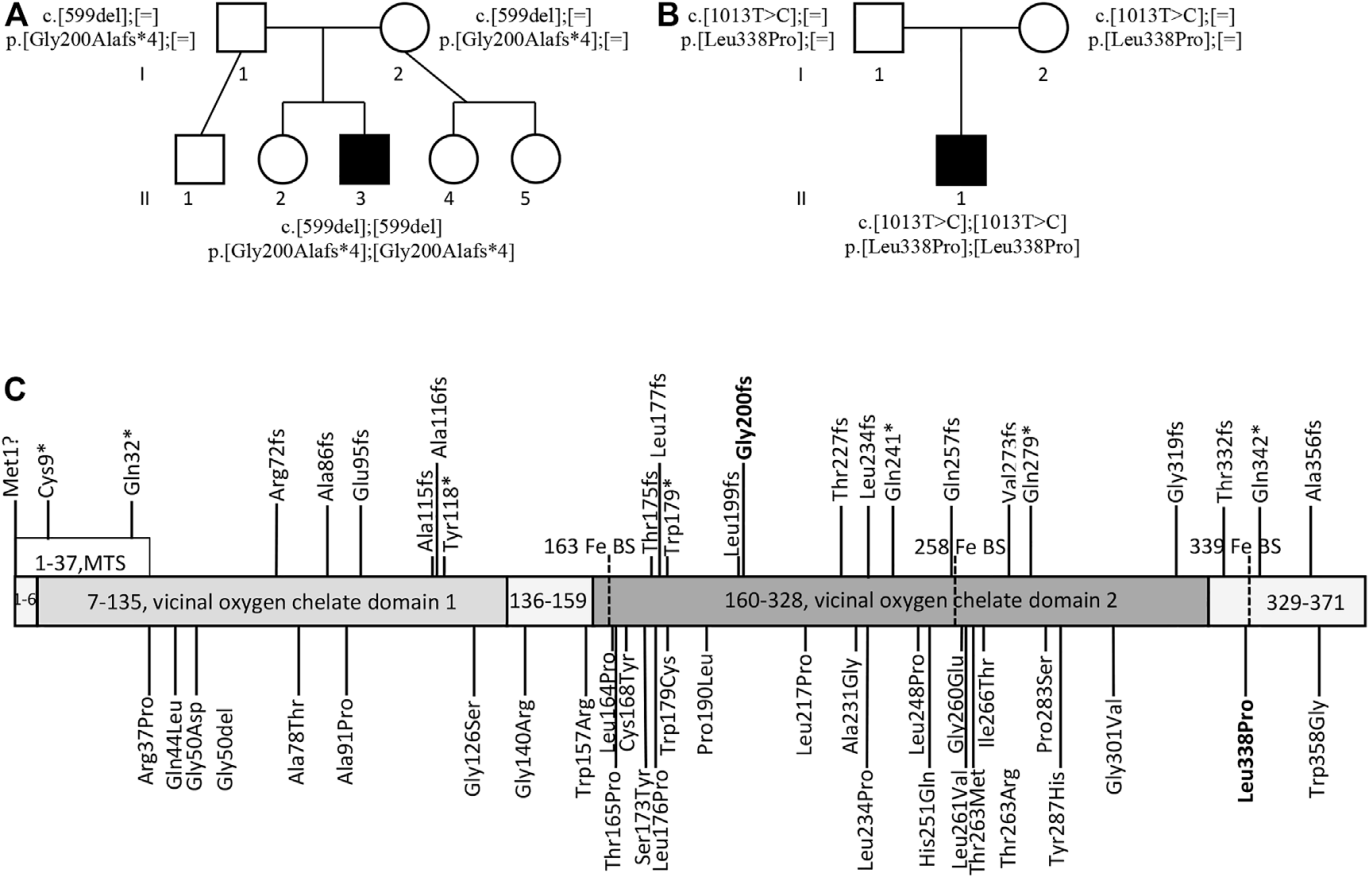
The probands’ pedigrees and HPDL variants. Pedigree of proband 1 **(A)** and proband 2 **(B)** with the harboured genotypes. Schematic of the HPDL protein and different disease-associated variants published so far **(C)** (Ghosh et al., 2020; Husain et al., 2020; Morgan et al., 2021; Numata-Uematsu et al., 2021; Sun et al., 2021; Wiessner et al., 2021; Yu et al., 2021). MTS, mitochondrial targeting sequence; Fe BS, iron binding site; variants reported in this manuscript are indicated in bold.

The pedigrees of both patients with the detected genotypes and a schematic of the HPDL protein with all the mutations reported so far in the *HPDL*-related disease patients (Ghosh et al., 2020; Husain et al., 2020; Morgan et al., 2021; Numata-Uematsu et al., 2021; Sun et al., 2021; Wiessner et al., 2021; Yu et al., 2021) is provided in the Figure 2.

### Putative Effect of c.1013T > C p. (Leu338Pro) on HPDL Protein Function

**FIGURE 3:**
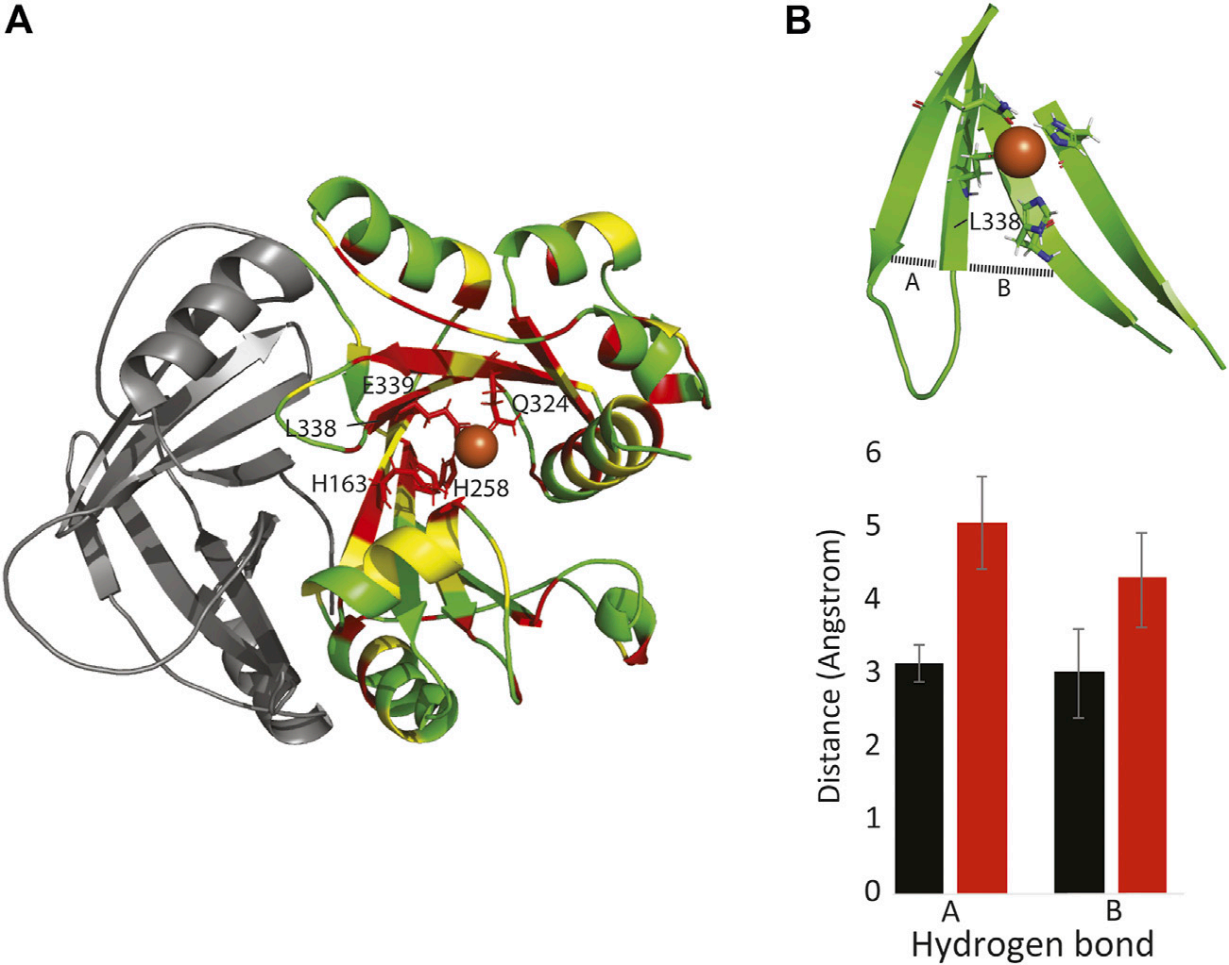
The Leu338Pro mutation disrupts HPDL structure and function. **(A)**: Model of one subunit of HPDL, based on the HPPD structure. Identical residues between HPPD and HPDL are colored red, similar residues are colored yellow. The N-terminal dimerization domain is colored in gray. Note that the four iron-binding residues are conserved between the two proteins, as are most residues surrounding the putative iron binding site. **(B top)**: HPDL Leu338Pro model showing how the beta strands that neighbor Pro388 dissociate. **(B bottom)**: This separation is due to a lack of inter-strand hydrogen bonds involving Pro338, along with missing hydrogen bonds between residues 327-337 **(A)** and 327-257 **(B)**.

To probe the effect of the c.1013T > C p. (Leu338Pro) mutation on HPDL protein structure and function, we first made a model of human HPDL based on the rat HPPD structure (pdb 1SQI) (Yang et al., 2004). Importantly, most residues in the central 7-stranded beta sheet are identical or similar to HPPD, including residues H163, H258, Q324, and E339 that coordinate an iron atom and are critical for HPPD enzymatic function (Moran, 2005). These four iron-coordinating residues lie across four of the central beta strands (Figure 3A).

Molecular dynamic simulations, run on both the HPDL and the HPDL (L338P) models, suggest that the mutation does not directly disrupt iron binding, but instead indirectly alters iron binding by disrupting the surrounding beta sheet. In particular, the substitution for the proline residue ablates hydrogen bonds to both of the neighboring strands, and consequently the mutant beta strands frays apart at the end (Figure 3B). Given these simulations and the mutation’s clinical manifestation, it is likely that the mutation either entirely destabilizes the C-terminus of HPDL, or the mutation disrupts iron binding by positioning one or more of the coordinating residues too far away from the iron atom.

## Discussion

Previously reported probands with *HPDL* variants and early disease onset presented with either severe neonatal encephalopathy, with little or no psychomotor development, or with a somewhat milder clinical course, with mild to severe developmental delay and progression of spasticity. By contrast, adolescent-onset disease has not been associated with developmental issues (Ghosh et al., 2020; Husain et al., 2020). In probands with infantile presentation, where developmental milestones were achieved, motor regression, with or without cognitive decline, was reported at various ages ranging from 3 to 6 years. Our proband 1 showed motor regression with gait changes from 4 years old and cognitive decline was noted at 6 years old, whereas proband 2 has shown no significant developmental progression and, therefore, no regression.

Seizures are reported in most probands with early onset disease and can be the presenting feature, occurring as early as the first days of life, but more often during the first 6 months. The severity of epilepsy is highly variable, with some probands continuing to have uncontrolled seizures despite treatment with different medications, and some achieving continuous remission, with no antiepileptic therapy, as illustrated by our cases. The brain MRI changes in both children were consistent with the MRI features of other probands with severe or intermediate course *HPDL*-associated disease.

A less recognized feature in initial reports of this syndrome was ataxia. Mild gait ataxia was noted in only one previously reported proband with a juvenile onset disease. By contrast, our proband 1 developed signs of cerebellar involvement at age 6–7 years, when the progressive disease course led to a more notable regression in motor and cognitive abilities. At that time, he demonstrated nystagmus, dysarthria, scanning speech, intention tremor, and dysmetria, as well as marked gait ataxia, which clearly contributed to his loss of ambulation. His cerebellar symptoms have been stable over time; however, the leg ataxia can no longer be demonstrated because of pronounced muscular hypotrophy and weakness of the lower extremities. His ocular and upper limb ataxia symptoms are demonstrated in the video supplement. We have not been able to elucidate any other possible causes of this feature from his genome sequencing data; however, the most recent proband compilation, including 34 new probands, reports this symptom as frequent in those with intermediate disease severity (Wiessner et al., 2021). A detailed table of clinical symptoms of both our probands in comparison with previously published patient compilations is included in the supplement. (Synofzik and Schüle, 2017) proposed modular phenotyping, introducing the concept of the ataxia-spasticity disease spectrum. In favor of this concept is the vast number of diseases with clinical and pathophysiological overlap between ataxias and hereditary spastic paraplegias, and the fact that attempts to develop a classification system suited to all of them have failed to date (Synofzik and Schüle, 2017). NGS techniques have allowed identification of causes of ataxia and hereditary spastic paraplegia as variants in the same genes and genetic pathways, and there are increasing numbers of articles describing the expansion of phenotypes and discussing a phenotypic continuum, as observed in probands with *HPDL*-associated disease.

The wide variability of the disease course observed in several probands, with a range of neurological symptoms and intermittent decompensation episodes, can itself be suggestive of mitochondrial disease. Nevertheless, only a fraction of affected children show biochemical signs of mitochondrial involvement, such as elevated lactate or alanine levels, or changes in respiratory chain complex activities. Analysis of mitochondrial OXPHOS complex activity in both our probands showed reduced citrate synthase and inconsistent reductions in complex I activity. It remains unclear whether the reduction of citrate synthase reflects a decrease in cellular mitochondrial content or an effect of the HPDL pathway on citrate synthase itself.

To date, little is known about the function of the HPDL protein. Sequence similarity with HPPD, especially surrounding the putative iron binding site, suggests the enzyme is an iron-dependent oxygenase, however the natural substrate remains unknown.

HPDL contains a mitochondrial targeting signal and localizes in the outer mitochondrial membrane (OMM). OMM proteins are involved in fission and fusion of mitochondria, referred to as mitochondrial dynamics (Xian and Liou, 2021), as well as in apoptosis, where OMM permeabilization is considered a “point of no return” during this process (Chipuk et al., 2006). Possible involvement of HPDL in apoptosis was revealed by Ghosh et al., who reported that Hpdl knock-out mice exhibit apoptosis in the brain, together with neurological regression and small brain size (Ghosh et al., 2020). The effect of HPDL depletion on mitochondrial fission and fusion in HeLa cells was studied by Wiessner et al., and did not result in altered cell oxygen consumption or mitochondrial dynamics (Wiessner et al., 2021). Nevertheless, it is possible that the lack of mitochondrial dynamics pathology can be explained by the differing sensitivities of various cell types to HPDL depletion, as supported by evidence from Hpdl knock-out mice, in which brain neurons showed more evidence of apoptosis than glial cells (Ghosh et al., 2020). Further studies are required to elucidate the effect of this HPDL on mitochondrial survival and metabolism, and its implications for disease pathogenesis.

## Data Availability

The datasets presented in this study can be found in online repositories. The names of the repository/repositories and accession number(s) can be found below: https://databases.lovd.nl/shared/individuals/00383056, 00383056; https://www.ncbi.nlm.nih.gov/clinvar/variation/1328237,1328237; https://www.ncbi.nlm.nih.gov/clinvar/variation/1327472,1327472.
