# Supplementary material for: Case Report: Two Families With HPDL Related Neurodegeneration
Source: Front Genet. 2022 Feb 9;13:780764. doi: 10.3389/fgene.2022.780764 (PMC8864118; doi:10.3389/fgene.2022.780764)
Supplement: Supplementary file 3 [file Table1.DOCX]

**Methods**

*Recruitment of families and ethics statement*

Persons affected with rare unidentified inherited disease have been recruited for inclusion in the Genome Database of Latvian Population (Riga, Latvia) under the framework of the Latvian Research Council project No: lzp-2018/1-0180 “The characterization and analysis of mitochondrial DNA mutations and variants of unknown significance using transmitochondrial cytoplasmic hybrid cell models”. Central Committee of Medical Ethics of Latvia approval (protocol No. 2019-3, chapter 7, from 30.05.2019.) covers all consent and data handling related issues for genetic research into the patients involved. Persons affected with rare neuromuscular, neurodegenerative, metabolic, or poly-malformation syndromes have been recruited in an interdisciplinary research program designated "Programme de Recherche et Innovation Sur les Maladies rarES" (PRISMES) at the CHU de Québec - Université Laval (CHUQC-UL) Research Centre. Research ethics board approval of the study design was obtained from the Comité d’éthique à la recherché (CER) du CHUQC-UL.

All participants and/or their legal guardians provided written informed consent prior to enrolment. The parents of the patient 1 have specifically consented to the use of video files for publication.

*DNA extraction*

Blood samples were drawn from recruited individuals. Total DNA was extracted from peripheral blood leukocytes using the standard phenol/chloroform method for samples associated with patient 1 and a QIAamp DNA Blood kit (Qiagen, Toronto, ON, Canada), according to the manufacturer’s instructions, for samples associated with patient 2.

*Library preparation and whole genome sequencing*

Libraries were prepared from 300 ng of high-quality genomic DNA using a MGIEasy Universal DNA Library Prep Set (MGI Tech Co., Shenzhen, China), according to the manufacturer’s instructions. Libraries were sequenced at a mean coverage of 30× on the MGI MGISEQ-2000RS platform, using a nanoball approach, to generate 150 bp paired-end reads.

*Library preparation and whole exome sequencing*

Libraries were prepared from 3 µg high-quality genomic DNA using a SureSelect XT human All exon V6+UTR kit (Agilent Technologies, Santa Clara, USA), according to the manufacturer’s instructions. Libraries were then subjected to exome capture. Three libraries with unique indices were pooled together in an equimolar ratio and sequenced at a mean coverage of 100× on the Illumina HiSeq2500 platform to generate 125 bp paired-end reads.

*Bioinformatics analyses of genome data and variant filtering, patient 1*

The fastq files were processed and variants were called using GATK v4 best practice guidelines using wdl scripts and Cromwell workflow management system [1]. Shortly, raw sequencing reads were mapped to hg38 reference genome using BWA MEM algorithm (v 0.7.15) [2], followed by the processing using GATK (v4.0.6.0) including flagging duplicated reads and base score. Variants were first called for individual samples using GATK HaplotypeCaller, before multi-sample joint aggregation and re-annotation using GATK GenotypeGVCFs. The variant call set was annotated using hail 0.1 (Hail Team. Hail 0.1. https://github.com/hail-is/hail) and then uploaded to the Seqr platform (https://github.com/broadinstitute/seqr) for collaborative variant analysis. Variant filtration focused on rare variants in coding regions and splice-sites predicted to affect protein function and following the known inheritance pattern for the disease associated genes.

*Bioinformatics analyses of exome data and variant filtering, patient 2*

Data were processed using a pipeline adjusted from GATK Best Practices and the Snakemake workflow (https://github.com/snakemake-workflows/dna-seq-gatk-variant-calling). Raw data were demultiplexed using proprietary Illumina bcl2fastq software to generate open format data. Then, raw reads were trimmed using Trimmomatic [3] and mapped to the human reference genome (hg19) using BWA [2]. Duplicated reads were flagged using Picard MarkDuplicates (https://broadinstitute.github.io/picard/) and base score recalibration was performed using GATK BaseRecalibrator [4]. Variants were first called for individual samples using GATK HaplotypeCaller, before multi-sample joint aggregation and re-annotation using GATK GenotypeGVCFs. Variants were functionally annotated based on data from SiFT [5], CADD [6], avsnp, Kaviar, ExAC, esp6500siv, 1000 genomes, and Polyphen 2 [7], using Annovar, VEP [8], and the SEQR platform (https://github.com/broadinstitute/seqr).

*Mitochondrial respiratory chain complex analysis in peripheral blood leukocytes*

Peripheral blood samples (10–20 ml) were collected and leukocytes were isolated by centrifugation (600 x g, 10 min, room temperature) in RBC lysis buffer, and then washed with PBS. Isolated leukocytes were disrupted by homogenization with a Dounce homogenizer in medium containing 0.1 mg/ml digitonin, as described previously [9]. Obtained mitochondria were suspended in buffer containing 25 mM KH2PO4 and 5 mM MgCl2 (pH 7.2). Respiratory chain complex I–IV and citrate synthase activities were measured spectrophotometrically, as described previously [10], with modifications. Four technical replicates were conducted for each patient’s sample. The control group consisted of 17 healthy volunteers (age 23–65 years, mean 35.5 years).

*Molecular dynamics simulations*

Computational modeling was completed using HPPD (PDB: 1SQI) as a template [11]. The human HPDL sequence was threaded through this original structure using Phyre2 [12] and was then allowed to equilibrate in the computer program YASARA 20.8.1 using the Amber ff14 forcefield as previously described [13] until the backbone RMSD no longer changed significantly (~15 ns) [14]. The Leu338Pro substitution was then engineered via the ‘swap’ command, and the resulting model was monitored for ~150 ns in triplicate. Analysis was conducted in YASARA, and was compared to similarly-run simulations involving the WT HPDL model.

References

1 – Van der Auwera G. A., O'Connor B.D. Genomics in the Cloud: Using Docker, GATK, and WDL in Terra 1st Edition (O'Reilly Media, 2020).

2 – Li H., Durbin R. Fast and accurate long-read alignment with Burrows–Wheeler transform, Bioinformatics, 26(5) 589–595 (2010) https://doi.org/10.1093/bioinformatics/btp698

3 – Bolger A. M., Lohse M., Usadel B. Trimmomatic: a flexible trimmer for Illumina sequence data. Bioinformatics 30(15) 2114-2120 (2014) doi: 10.1093/bioinformatics/btu170

4 – McKenna A. et al. The Genome Analysis Toolkit: a MapReduce framework for analyzing next-generation DNA sequencing data. Genome research 20(9) 1297–1303 (2010) https://doi.org/10.1101/gr.107524.110

5 - Kumar P., Henikoff S., Ng P.C. Predicting the effects of coding non-synonymous variants on protein function using the SIFT algorithm. Nat. Protoc. 4(7) 1073-81 (2009) doi: 10.1038/nprot.2009.86.

6 - Kircher M.et al. A general framework for estimating the relative pathogenicity of human genetic variants. Nat. Genet. 46(3) 310-5 (2014) doi: 10.1038/ng.2892

7 - Adzhubei I., Jordan D. M., Sunyaev S. R. Predicting functional effect of human missense mutations using PolyPhen-2. Curr. Protoc. Hum. Genet. Chapter 7:Unit7.20. (2013) doi: 10.1002/0471142905.hg0720s76.

8 - Wang K., Li M., Hakonarson H. ANNOVAR: functional annotation of genetic variants from high-throughput sequencing data. Nucleic Acids Res. 38(16) e164. (2010) doi: 10.1093/nar/gkq603.

9 – Ma, Y. Y. et al. Analysis of the mitochondrial complex I-V enzyme activities of peripheral leukocytes in oxidative phosphorylation disorders. J. Child Neurol. 26(8) 974-9 (2011) doi: 10.1177/0883073811399905

10 – Feichtinger, R. G. et al. Low aerobic mitochondrial energy metabolism in poorly- or undifferentiated neuroblastoma. BMC Cancer 10 149 (2010). doi: 10.1186/1471-2407-10-149

11 – Yang C. et al. Structural basis for herbicidal inhibitor selectivity revealed by comparison of crystal structures of plant and mammalian 4-hydroxyphenylpyruvate dioxygenases. Biochemistry 43 10414-23 (2004) doi: 10.1021/bi049323o

12 – Kelley L. A., Mezulis S., Yates C. M., Wass M. N., Sternberg M. J. The Phyre2 web portal for protein modeling, prediction and analysis. Nat. Protoc. 10(6) 845-58 (2015) doi: 10.1038/nprot.2015.053

13 – Ackermann M. A. et al. Small Obscurins at the Intercalted Disc Mediate Cardiomyocyte Adhesion and Size via the PI3K/AKT/mTOR Pathway. J. Cell. Cardiol. 111 27-39 (2017) doi: 10.1016/j.yjmcc.2017.08.004

14 – Krieger E., Vriend G. YASARA View - molecular graphics for all devices - from smartphones to workstations. Bioinformatics 30(20) 2981-2 (2014) doi: 10.1093/bioinformatics/btu426
