# Supplementary material for: Case Report: Two Families With HPDL Related Neurodegeneration
Source: Front Genet. 2022 Feb 9;13:780764. doi: 10.3389/fgene.2022.780764 (PMC8864118; doi:10.3389/fgene.2022.780764)
Supplement: Supplementary file 4 [file Table2.DOCX]

Supplementary Figure 1.

Analysis of mitochondrial respiratory chain complex in peripheral blood leukocytes from patient 1. Four technical replicates were conducted. The control group consisted of 17 healthy volunteers (age 23–65 years, mean 35.5 years). CS: citrate synthase, CI: complex I, CII: complex II, CIII: complex III, CIV: complex IV.
